# P-454. The Skip Phenomenon in Staphylococcus aureus Bacteremia: Prevalence in a Pediatric Tertiary Care Center

**DOI:** 10.1093/ofid/ofaf695.669

**Published:** 2026-01-11

**Authors:** Mitchell J Witkowski, Muayad Allali, James B Wood

**Affiliations:** Emory University School of Medicine, Atlanta, GA; Indiana University School of Medicine, Indianapolis, Indiana; Indiana University School of Medicine, Indianapolis, Indiana

## Abstract

**Background:**

Bacteremia due to *Staphylococcus aureus* infections has been well-documented to exhibit fluctuating positivity in blood cultures, known as the “skip phenomenon” (SP). This phenomenon is defined as having at least one negative blood culture occurring between two positive cultures during a single episode of bacteremia while on adequate antibiotic therapy. However, data on its prevalence in pediatric patients remains scant. Current Infectious Diseases Society of America (IDSA) guidelines do not clearly define the number of negative culture sets needed to determine bacterial clearance. Common practice has been to obtain at least two serially negative blood cultures, but this is primarily based on adult data and observations. This study sought to assess the prevalence of the SP in pediatric patients with central lines and *S. aureus* bacteremia at a tertiary pediatric referral center.

**Figure:**
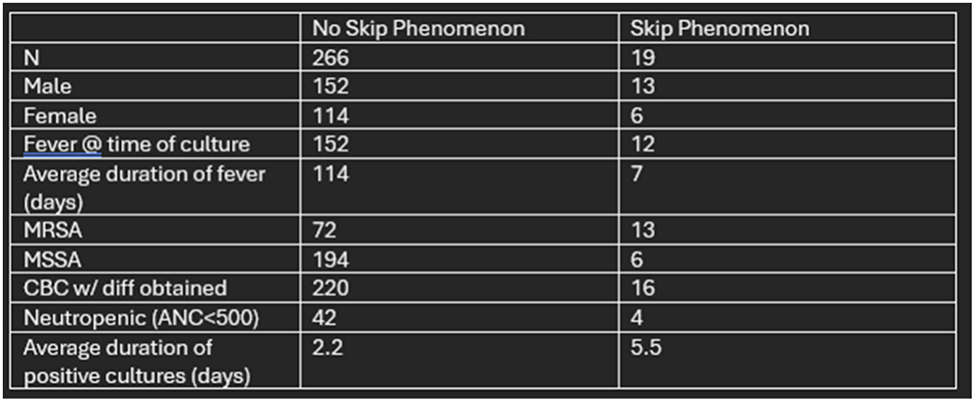

**Methods:**

We conducted a retrospective review of pediatric admissions from January 2011 to February 2024 with a *S. aureus* associated central line infection. Data collected included antimicrobial resistance patterns, duration of bacteremia, absolute neutrophil count (ANC), and blood culture history.

**Results:**

Among 285 patients with *S. aureus* CLABSI, 19 (6.6%) exhibited the SP. Of these 19 cases, 13 (68%) were due to methicillin-resistant *S. aureus* (MRSA), although MRSA accounted for only 30% of the total cohort. 13/85 (15%) infections caused by MRSA demonstrated the SP versus 6/200 (3%) of MSSA infections. This means MRSA patients were approximately five times more likely to experience the SP compared to MSSA cases. Most notably, there was an increased average duration of positive cultures in the SP group (5.5 days) compared to those exhibiting no SP (2.2 days).

**Conclusion:**

Our findings support the practice of obtaining at least two consecutive negative blood cultures drawn at least 24 hours apart to confirm clearance of *S. aureus* bacteremia in pediatric patients. The skip phenomenon appears particularly common among patients with MRSA highlighting the need for vigilance when this organism is responsible.

**Disclosures:**

James B. Wood, MD, MSCI, Karius: Grant/Research Support|MeMed: Grant/Research Support

